# Feasibility of online learning among Indian students of dentistry during the CoVid-19 outbreak

**DOI:** 10.6026/973206300200362

**Published:** 2024-04-30

**Authors:** Sonal Saigal, Narendra Nath Singh, Ankur Bhargava, Swati Singh, Lokesh Tomar, Somya Salwi

**Affiliations:** 1 Department of Oral Pathology, Microbiology & Forensic Odontology, Dental Institute, RIMS, Ranchi - 834009, India; 2 Department of Oral Pathology & Microbiology, Hazaribag College of Dental Sciences & Hospital, Hazaribag - 825301, India; 3 Department of Pediatric and Preventive Dentistry, Hazaribag College of Dental Sciences & Hospital, Hazaribag - 825301, India; 4 Delhi Institute of Health Care and Research, New Delhi - 110087, India

**Keywords:** COVID-19, coronavirus, education, dental, dentistry

## Abstract

Even though there were several online dentistry academic programs available, the pandemic accelerated the development of e-learning processes and presented unprecedented obstacles to dental education. The pandemic has given rise to a technology-powered teaching style that replaced the centuries-old chalk-talk method. However, because it was a quick change, it had flaws and limitations and has caused turmoil and confusion among many educational teams, particularly in the academic sector. As a necessary consequence, this study is performed to evaluate undergraduate dental students' lived experiences, as well as their capability, willingness and frame of mind for the adoption of online teaching and learning approaches as part of blended learning. Hence, the use of online tutorials should be an effective method of providing meaningful insights for undergraduate dental students.

## Background:

The CoVid-19 pandemic has necessitated the implementation of social distancing measures, which have had adverse effects on multiple aspects of society. One area significantly impacted is the education system, which plays a vital role in a nation's economic progress. On February 11, 2020, the World Health Organization (WHO) suggested labeling the virus as COVID, an abbreviation for corona virus disease [1]. It upset the lives of all and disrupted education systems around the world. Society was in the "hallucinating stage" at the beginning of the pandemic and believed that things would return to "normal" in the fall [2]. CoVid-19 challenges global educational organizations, prompting governments to rapidly transition from face-to-face to online and virtual classes, highlighting the need for significant progress [3]. There are several e-learning platforms that allow teachers to interact with their students, and in some cases, national television and social media platforms are used for educational purposes. Many schools have announced long vacations to prepare for this distance learning scenarios [4]. Online learning is now the sole choice to lead with the semester amid the lockdown and social distancing era. Implementing a well-structured "blended" preclinical curriculum and integrating knowledge and skills in order to produce better-trained doctors is highly recommended [5]. The spread of CoVid-19 has also caused fear, uneasiness, and various concerns among citizens around the world. When extended to circumstances caused by social restriction and other individual components, it is expected that parents' concerns were influenced by their inability to support their children in distance/online learning, the need to access vital innovations and the web, or the lack of innovative designs used for children with special educational needs and financial problems [6]. Therefore, it is of interest to record and collect experiences through a data collection process on students' possibilities, adequacy and views about online education and learning.

## Materials and Method:

## Study setting:

This study was conducted for BDS (1st to final year) students across India, a self-administered questionnaire survey that was created, designed, and disseminated using the Google Forms platform. Participants were encouraged to fill out the form and assist in sharing the questionnaire with their BDS friends. Thus, participants were recruited by a snowball sampling technique. Moreover, participants were limited to one response to avoid duplicated or exaggerated data.

## Data collection:

The questionnaire had four sections. The first section included an introduction to the research that emphasized the confidentiality of the questionnaire. The second section was about the consent of the respondent (Item 1). The third section was diversified in demographic profiles like age, gender, study level, name of the Institute, zone of India, etc. (Items 2-8).The fourth section of the questions covered the level of feasibility, acceptability, and attitudes of students related to online learning during the CoVid-19 outbreak (Items 9-15).The validity of constructs was evaluated by a professional practitioner heavily involved in public health. A pilot study was conducted to assess the reliability of the questionnaire.

## Sample size:

The formula for calculating the sample size can be expressed as follows: E=sqrt ((Z^2*p*(1-p))/n), where, Z=Z-value for the desired confidence level, p represents the estimated proportion or expected prevalence, and E represents the margin of error. By substituting the given values into the formula: E = sqrt ((1.96^2 * 0.5 * (1 - 0.5)) / n), the result is E = 0.098. Therefore, with a desired sample size of students and assuming a 95% confidence level, the margin of error would be approximately 9.8%. It is important to note that this sample size calculation assumes the use of a simple random sampling technique and a large population size. Nonetheless, the survey managed to gather participation from a total of 700 dental students representing various dental institutes across India. Participants were stratified into 2 categories (Undergraduate Dental Students aged 18-21 years). Based on these criteria we revived 700 responses across the country from 4 different regions of India.

## Methodology:

In the present questionnaire survey, national wide volunteer students from BDS first - final year were included. This study aimed to maximize reach and gather data from as many respondents as possible. Additionally, a combination of purposive and snowball technique was used to select the respondents via social media platforms for capturing data. The questionnaire was only for undergraduate Dental students (B.D.S). Anonymity and confidentiality of answers were assured to all students, and they were entitled to answer freely and without fear. They were also assured that the information given by them is for research and evaluation purpose only and will be confidential. Online informed consent was obtained before proceeding with the questionnaire. Questions for the survey were developed after reviewing the published literature, and the validity of the questionnaire is established by obtaining valuable opinions from subject experts. Once the questionnaire has submitted the responses were collected as excel sheets and were statically analyzed using appropriate tools with techniques like percentages, mean, standard deviation, chi-square, T-test, ANOVA.

## Results:

Several demographic variables were examined, including age, sex, education, and place of residence. Among the respondents, the mean age was 21 years. 700 undergraduate responses were received from all over India. The distribution of participants across different years of study revealed that a significant portion, 34.6%, belonged to BDS 1st year, followed by 25.8% in the BDS 2nd year, 20.9% in the BDS 3rd year, and 18.7% in the final year, as depicted in Figure 1 (see PDF). In terms of gender, a considerably high percentage, 77.2%, of female participants was observed. Most of the respondents were belonging to the rural background 140 (45.60%) whereas 121 (39.41%) were from urban areas and only 46(14.98%) were from peri-urban areas. The geographic distribution of participants in the study illustrated that the majority (44.6%) hailed from the northern region of India as shown in Figure 2 (see PDF) and out of these, 66% belonged to a private set-up. The data were analyzed utilizing SPSS 22.0, with the application of chi-square tests on qualitative data to establish hypotheses at a significance level of 0.05. The data were scored on a Likert scale, and descriptive statistics were used to express the data, such as frequency and hypotheses. Based on the findings, there was a highly significant association between age, gender, qualification, set-up, and location.

After analyzing numerous responses, a substantial proportion of participants indicated their concurrence that online education saves time. However, they encountered difficulties due to insufficient technical support, particularly related to network connectivity issues in their residential areas. Furthermore, unscheduled power outages disrupted their online classes. Despite receiving helpful study materials, they expressed dissatisfaction with the resolution of their doubts, as it did not match the level of support; they were accustomed to receiving in offline classes. Overall adapting to this new method of online learning is challenging for them leading to a decrease in motivation.

## Subject-wise satisfaction level:

The participants in the first and second years of BDS expressed a moderate level of disagreement in theory classes, regarding the effectiveness of online classes in helping them achieve their learning objectives for various subjects.

The practical classes of Oral Pathology and Preclinical Prosthodontics Crown & Bridge showed clear instances of strong disagreements. Similarly, the respondents also expressed notable levels of disagreement regarding the achievement of learning goals in the subjects of Biochemistry, Dental Anatomy, and Dental Histology (DADH), both in theory and practical classes.

Among BDS 3rd and Final-year students, Students agreed that they were able to learn and achieve the learning goals in theory subjects such as Oral Medicine, Orthodontics, Oral Pathology, Prosthodontics, Conservative Dentistry, Periodontics, Oral Surgery, General Surgery, General Medicine, and Public Health Dentistry. However, in the case of Pedodontics theory, they strongly disagreed regarding their ability to learn and achieve the learning goals.

A comparison between face-to-face learning and online learning was conducted among third and final-year dental students, considering their clinical skills. During the pandemic, 30.7% of the students reported that their institution did not suspend their clinical training/posting. However, it is important to note that the patient flow for dental treatment experienced a significant decline. On the other hand, 11.4% of the students confirmed that their institution did suspend their clinical training/posting. The findings revealed a decrease in confidence when it came to interacting with patients. In our study, 38.1% of the participants expressed a preference to postpone their training/course, indicating a hesitation to proceed with their clinical education. Additionally, 7.3% of the students lacked confidence in their skills and the acquisition of solid clinical knowledge prior to graduation. Approximately 48.7% of BDS students expressed significant dissatisfaction with communication, while 46.3% reported dissatisfaction with the learning style, and 46% indicated dissatisfaction with interaction as indicated in Figure 3 (see PDF).

## Discussion:

In developing countries, the online learning system is relatively uncommon and considered a newer approach. The findings of this study indicate that a majority of students feel that they are not equally challenged in an online class and do not experience the same level of learning as they would in traditional classroom settings This finding is consistent with previous research done by Gopal R *et al.* (2021) [7]. Regarding the research question in our study, most participants voiced their discontent with online instruction. More specifically, 46% of the participants believed that online teaching lacks interactivity, while 48% perceived communication effectiveness as low, and 46.3% expressed disappointment with the learning styles. Moreover, participants widely agreed that unscheduled power cuts and insufficient technical support, such as network problems in their residential areas, impeded their online learning experience. Several studies conducted during the pandemic have shown a widespread negative perception of online learning among students. Abbasi *et al.* (2020) [8] found that 77% of participants held a negative attitude toward online learning, while Agormedah *et al.* (2020) [9] reported a negative perception by 43.3% of participants. Angung *et al.* (2020) [10] found that 66.7% of participants held a negative perception, and Nugroho *et al.* (2020) [11] observed that 82% had a negative attitude toward online courses. Susilana *et al.* (2020) [12] discovered that 71.6% of participants found online learning to be more challenging than face-to-face instruction. Additionally, in a study by Ebohon *et al.* (2021) [13], a significant percentage of students (67%) and teachers (59%) acknowledged limited interactions, which negatively impacted student satisfaction. These findings resonate with our study, indicating a shared observation of a negative perception of online learning among students.

According to the studies conducted by Badovinac *et al.* (2021) [14], Varvara G *et al.* (2021) [15], Bisht *et al.* (2022) [16], and Kapasia *et al.* (2020) [17], the effects of lockdown on students' learning performance were examined. The findings revealed that the lockdown had a significant negative impact on students' learning experience, resulting in various challenges such as increased anxiety and depression, difficulties with internet access, and an unfavorable home learning environment. Marginalized and remote students faced significant challenges, similar to those identified in other studies. These challenges include limited access to technology, lack of social interaction, insufficient practical training, and difficulties with interactive learning in medical education. This reinforces the effectiveness of our approach and adds further evidence to support our findings [14-18].

Recent studies conducted by Petre *et al.* (2023) [19] provide further support for the methodology proposed in our study and validate students' positive reception of e-learning or virtual teaching. The authors note that e-learning facilitates convenient access to shared materials, enhancing students' comprehension of intricate clinical cases or theoretical subjects, aligning with our findings.

In contrast to our study, Petre *et al.*'s research emphasizes the importance of increased interaction between students and their peers as well as instructors. This finding aligns with the observations made in various previous studies, including the research conducted by Khalil *et al.* (2020) [20] and Annamalai *et al.* (2022) [21].

Some studies have placed emphasis on the role of gender in the adoption of online education, specifically noting that female students demonstrated a stronger preference for online learning in terms of assignments and study patterns [22, 23]. However, it is important to note that in our own research, although most participants were female, we did not prioritize gender or conduct any assessments based on gender related differences.

Studies reveal contrasting results on student satisfaction and stress levels during online teaching-learning. Male students express higher satisfaction levels, while female students report higher stress during the CoVid-19 pandemic. Male students show greater learning interest, attention, attendance rates, and overall satisfaction, possibly due to their computer familiarity and ease of adapting to online teaching methods [24, 25, 26].

This research stands out for its comprehensive analysis of dental subjects, encompassing both theoretical and practical aspects, with a specific focus on evaluating the efficacy of online learning in the context of the CoVid-19 pandemic. By addressing this research gap, the study aims to offer valuable insights into the effectiveness and feasibility of online dental education during these unprecedented times. Data indicate significant disagreements in practical classes, specifically in Oral Pathology and Preclinical Prosthodontics Crown & Bridge. The lack of satisfaction with online instruction in certain subjects, such as those with a hands-on nature like Biochemistry, Dental Anatomy, and Dental Histology (DADH), suggests that students may have felt that online learning was inadequate for acquiring practical skills and experiences. This sentiment was expressed both in theory and practical classes. These findings underscore the significance of practical and laboratory-based learning experiences in dental education and the need for alternative approaches to ensure effective learning in these subjects.

Among third and final-year BDS students, online instruction was generally perceived positively in several theory subjects, including Oral Medicine, Orthodontics, Oral Pathology, Prosthodontics, Conservative Dentistry, Periodontics, Oral Surgery, General Surgery, General Medicine, and Public Health Dentistry. This suggests that online learning was effective in helping students achieve their learning goals in these areas. However, there was disagreement among students when it came to the subject of Pedodontics theory, possibly indicating that this particular subject needs improvement in online delivery. It is worth noting that there is a lack of existing studies that specifically examine the challenges and opportunities of online learning in the dental field, considering both theoretical and practical aspects across all subjects, during these unprecedented times. Therefore, this research provides valuable insights into the effectiveness of online instruction in dental education, highlighting both the areas of success and areas that require further attention.

## Limitations of the study:

The lack of a control group in online learning studies makes it difficult to attribute any differences in perceptions solely to online learning. There are external factors such as personal circumstances, technological limitations, or institutional support that may also have shaped the students' perceptions, which the study may not have considered. Therefore, it is crucial for researchers to acknowledge these limitations and consider them when interpreting the study's results, which may not be applicable to students in other healthcare disciplines or educational settings.

## Recommendations for the new normal in dental education and e-learning strategies include:

[1] Real-time communication platforms and interactive multimedia content could enhance engagement with dental concepts and overcome face-to-face instruction. It may help bridge communication gaps and improve dental education.

[2] Continuous evaluation and improvement of e-learning strategies, student feedback, and adjustments that may enhance online dental education should be provided. Tracking student progress and evaluating student performance using online assessments is also crucial. A variety of assessment methods can be used to measure theoretical knowledge and clinical skills, including virtual, case-based, and objective structured clinical examinations (OSCEs).

[3] It is essential to prioritize faculty development, training, and the adoption of technology to effectively deliver online dental education. This includes honing professional skills and keeping abreast of emerging technologies to enhance student learning. By investing in faculty development and embracing innovative technologies, we can ensure that online dental education remains relevant, engaging, and aligned with the evolving needs of students.

## Unveiling the distinctive tapestry:

This research endeavor delved into uncharted territory by investigating different facets of dental students' difficulties, encounters, and educational achievements in an online learning setting. Throughout four years, dental students undergo hands-on clinical and practical instruction. Despite receiving clinical and practical training during the pandemic, dental students continue to face challenges. They acknowledged that online theoretical classes have proven to be more effective, but they still emphasized the importance of in-person practical implementation. Future research should focus on web-based methods, simulator usability, and educator preparation. Online instruction success relies on well-designed materials, motivating interaction, and well-organized teachers.
